# The Frozen Effect: Objects in motion are more aesthetically appealing than objects frozen in time

**DOI:** 10.1371/journal.pone.0215813

**Published:** 2019-05-16

**Authors:** Malerie G. McDowell, Jason Haberman

**Affiliations:** 1 Department of Psychology, Vanderbilt University, Tennessee, United States of America; 2 Department of Psychology, Rhodes College, Tennessee, United States of America; Universita degli Studi di Udine, ITALY

## Abstract

Videos of moving faces are more flattering than static images of the same face, a phenomenon dubbed the Frozen Face Effect. This may reflect an aesthetic preference for faces viewed in a more ecological context than still photographs. In the current set of experiments, we sought to determine whether this effect is unique to facial processing, or if motion confers an aesthetic benefit to other stimulus categories as well, such as bodies and objects—that is, a more generalized ‘Frozen Effect’ (FE). If motion were the critical factor in the FE, we would expect the video of a body or object in motion to be significantly more appealing than when seen in individual, static frames. To examine this, we asked participants to rate sets of videos of bodies and objects in motion along with the still frames constituting each video. Extending the original FFE, we found that participants rated videos as significantly more flattering than each video’s corresponding still images, regardless of stimulus domain, suggesting that the FFE generalizes well beyond face perception. Interestingly, the magnitude of the FE increased with the predictability of stimulus movement. Our results suggest that observers prefer bodies and objects in motion over the same information presented in static form, and the more predictable the motion, the stronger the preference. Motion imbues objects and bodies with greater aesthetic appeal, which has implications for how one might choose to portray oneself in various social media platforms.

## Introduction

Visual experience is one of change, wherein people and objects constantly move about their environments. Motion signals are so inextricably linked to our perception of the world, that the brain has evolved neural modules exclusively dedicated to processing motion information [1–3]. Given the ubiquity of motion in our visual experience, it is no surprise that the role of motion in object perception has been extensively explored (e.g., [4, 5]). In face perception, for example, there is general consensus that motion confers a benefit to the recognition of familiar faces, a phenomenon referred to as the ‘motion advantage’ (e.g.,[6–8]). It most prominently manifests itself when viewing degraded images (e.g., negatives; [6]), ostensibly because under such viewing conditions recognition systems cannot rely upon cues typically available in a static image [9]. The visual system depends upon ‘dynamic facial signatures,’ cues present in moving faces, to identify faces in suboptimal viewing conditions [10]. This benefit is not unique to the identification of faces—emotional expressions are perceived as more salient in faces that are moving [11], further supporting a crucial role for motion in evaluating facial properties. These robust motion detection systems appear to be in place at an early age, as even infants show differential eye-movement patterns to faces in motion versus their static counterparts [12].

While the motion advantage has been extensively explored, from the role of familiarity (e.g., [13]) to plausible neural mechanisms (e.g., [9]) to the development of critical dynamic facial signatures [10], relatively fewer studies have examined whether motion confers an analogous enhancement of aesthetic preference. For example, researchers showed that attractiveness judgments of artificial, computer-generated faces were enhanced by motion information alone[14]. Other work that has explored aesthetic preference differences between static and dynamic stimuli focused primarily on the ecological validity of using static images in behavioral research [15–17]. Penton-Voak and colleagues[15] found higher attractiveness ratings for dynamic stimuli (but only for videos depicting males) and overall marginal correlations between dynamic and static image ratings. In contrast, others found no mean differences in attractiveness ratings and strong and significant correlations between the ratings of static and dynamic face stimuli, leading those researchers to conclude that, while dynamic stimuli provide additional information not available in a static image, attractiveness is consistently assessed across both stimulus types [15, 16]. In these examples, however, observers evaluated just one, hand-picked static exemplar from the source video (or sometimes from a different source altogether), so they were not evaluating identical information content in both conditions.

Other recent work addressed this by having observers provide aesthetic ratings for *all* information within a given video for both static and dynamic conditions [18]. Researchers showed that pausing a video of someone speaking often produced a static image that was significantly less flattering than the video from which it came, a phenomenon authors called the ‘Frozen Face Effect.’ Source videos were of individuals speaking while directly facing the camera (without sound). Observers rated each video on a Likert scale of flattery (a proxy for attractiveness), as well as each individual video frame that composed the videos. Results showed an unequivocal preference for the faces in motion, mirroring the recognition benefit for faces in motion (although, unlike the results for recognition, the preference in the Frozen Face Effect did not depend on familiarity; [18]). Authors interpreted the results as reflecting the limitations of temporal integration, which essentially smooths over momentary deviations in facial expression that may be present in a static image (e.g., eye blinks) over the course of a dynamic stimulus.

If motion can improve the appearance of a face, does it also improve the appearance of other object categories, such as bodies? Indeed, previous work has stressed the importance of considering the role of body motion in judging properties like symmetry [19], gender [20], intention [21], and emotion [22]. Other research examining attentional effects of bodies-in-motion revealed differential impact on the deployment of attention depending on whether the stimulus was dynamic [23]. In the static condition, observers tended to focus on facial information to aid recognition, whereas in the dynamic condition, observers more equitably deployed attention across both body and face, and overall identification performance improved as a result. The reliance on the body for identification should come as little surprise when one considers the wealth of information provided even by simple, dynamic point light walkers, as referenced above (e.g., [20]).

Neuroaesthetics is a burgeoning area of research that attempts to link aesthetic experience with their underlying brain processes [24–26]. Recent work exploring the neuroaesthetics of dance have revealed compelling relations between movement rated as highly aesthetic and neural activity [24, 26]. Dance is a performance art; its appreciation is dependent wholly on the perception of bodies in motion. Authors identified enhanced activity in visual and sensorimotor areas in response to movements participants found most aesthetically appealing. The link between the aesthetic judgment of motion and their neural underpinnings points to a crucial role for motion in our experience of aesthetics.

In the current set of experiments, we build upon and extend the original Frozen Face Effect by examining aesthetic preferences for other categories of objects in motion, biological and non-biological. We asked observers to evaluate three categories of stimuli: Bodies making non-prototypical movements, bodies making prototypical movements, and non-biological objects making prototypical movements. Observers provided aesthetic judgments for both videos and the corresponding frames composing those videos. Because observers were exposed to identical image content in both the dynamic and static conditions, there should have been no difference in the ratings between the two conditions if motion was irrelevant. Instead, observers judged the videos to be more flattering than their constituent static images, regardless of stimulus category (e.g., body or object). This ‘Frozen Effect’ persisted across all control experiments. Interestingly, the effect was enhanced as the displayed movements became increasingly prototypical or predictable.

## Experiment 1A

Experiment 1 explored whether the original FFE extended to other object categories, in this case, bodies.

### Materials and methods

#### Participants

Eighteen undergraduate students from the Rhodes College community, age 18–22, participated in this experiment. All participants were naïve to the purpose of the experiment. Participants earned course credit or were paid $10 per hour as compensation. All participants gave informed consent and had normal or corrected-to-normal vision. This research, and all research described herein, was conducted in accordance with the Declaration of Helsinki and approved by the Institutional Review Board at Rhodes College.

#### Stimuli and design

**Fig 1:**
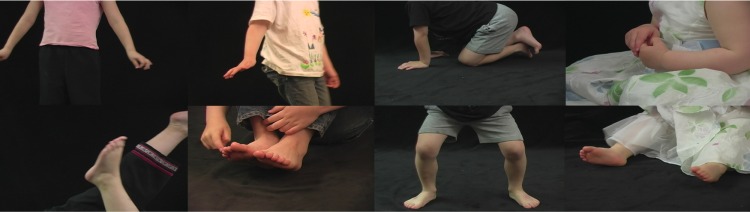
Examples of stimuli. All stimuli used were seen both in dynamic and static conditions. Reprinted with permission from Nancy Kanwisher.

Stimuli were 25 videos and their constituent still frames of children engaging in a range of movements, from subtle hand and foot movements to rapid jumping and waving (Fig 1). Videos were 1-second clips presented in the center of the screen at 30 fps at a resolution of 1280 × 720 pixels (subtending 30 × 16.5 degrees of visual angle). No faces were visible in the videos, isolating the role of bodily motion in observers’ evaluation, and also ensuring anonymity. All videos selected were shot from a stationary camera. The original video database was developed by Kanwisher and colleagues and has been used extensively as a category localizer for bodies in research utilizing fMRI (e.g., [27, 28]).

Within an experimental session, observers viewed both videos and their constituent 30 frames, presented in random order (and intermixed with other videos and static images). This ensured the information being evaluated in both conditions (motion and static) was identical.

#### Procedure

Observers were seated in a dimly lit, sound-dampened room with their heads resting on a chin rest positioned 63cm away from the display monitor. Observers viewed both the videos and the constituent images and were asked to evaluate how flattering a given depiction was on a scale from 1–7, with 7 being the most flattering—a rating system used previously by Post and colleagues[18]. Observers were told to assess flattery (as opposed to attractiveness) because it allows for variation in rating when viewing different depictions of the same body/object. For example, an individual might remain attractive even when depicted in an unflattering photograph—this procedure avoids that confound.

Static images remained on the screen until a response was received, followed by a 250 ms intertrial interval (ITI). Videos were played for one second (30 frames), without sound. The final frame remained on the screen until a response was received, followed by a 250 ms ITI. Each observer viewed a total of 25 videos and 750 static images (25 videos × 30 frames per video) for a total of 775 trials.

### Results and discussion

**Fig 2:**
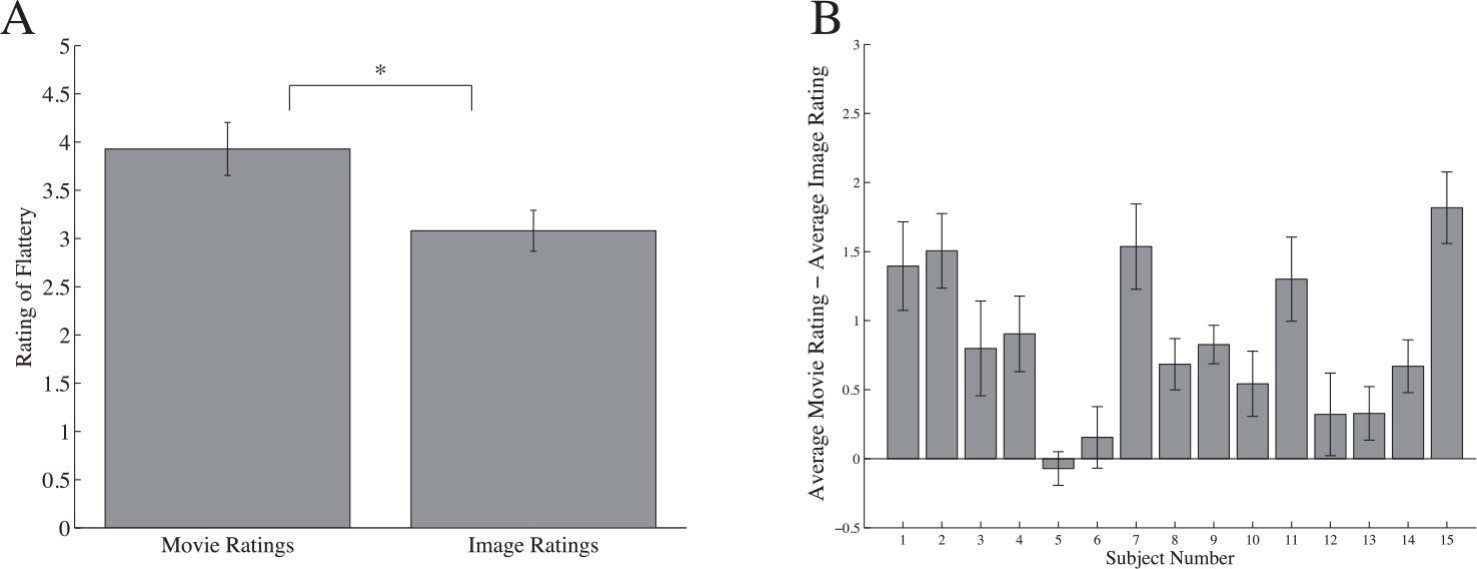
Flattery ratings. (A) The average flattery ratings of all movies compared to the average flattery ratings of all static images, collapsed across observers. Movie ratings were significantly higher than their component images. (B) The difference between the average of all movie ratings and the average of all static image ratings for each observer. Positive scores represent higher overall flattery ratings for bodies in motion. Error bars represent one standard error of the mean (SEM). Note: * denotes significance at p < .005.

Two participants were removed from analysis due to not following task instructions. For the 16 remaining participants, video ratings were compared to the average ratings of all constituent still frames. Besides continuous motion present in the videos, both conditions contained identical visual information—if motion did not impact flattery judgments, then the ratings across the two conditions should not differ. In fact, video ratings were significantly higher than the average rating of each video’s corresponding still images (t(15) = 6.34; p < 0.0001; *d* = 1.58; Fig 2A) suggesting flattery ratings were positively affected by the presence of continuous motion. This trend was true for nearly every observer, as shown in Fig 2B. These results indicate bodies engaged in motion are preferable to the same information displayed in static form.

As the last frame of the movie remained on the screen until a response was made by participants, we ran an additional control analysis to ensure participants were not basing their assessment of the movies on the last frame they viewed. If participants were not incorporating the rest of the sequence in the judgment, we would expect there to be no difference between the ratings of the movies and the ratings of the images corresponding to the last frames of the movies when presented in isolation. Our direct comparison of these ratings, however, revealed a robust Frozen Effect (*t*(14) = 6.26; *p* < 0.0001, *d* = 1.63), suggesting that observers were still evaluating the entirety of the movie even though they were exposed to the final frame for a longer period of time.

## Experiment 1B

Post and colleagues[18] found that the Frozen Face Effect persisted even when observers viewed inverted faces, suggesting that the FFE did not hinge upon holistic or configural information about the faces. In this experiment, we tested whether a similar pattern emerged with regard to bodies in motion. Given the relatively robust configural dependency found for bodies (on par with that of faces) [29], we might expect inverted bodies to reduce the magnitude of the Frozen Effect. However, we found an equivalent effect for both upright and inverted stimuli, similar to the original FFE, as described below.

### Method

#### Participants

Nineteen undergraduate students from the Rhodes College community, age 18–22, participated in Experiment 1B. All participants were naïve to the purpose of the experiment.

#### Stimuli and design

The videos and images were identical to those used in Experiment 1A, except they were displayed to observers inverted.

#### Procedure

The experimental procedure was identical to that of Experiment 1A.

### Results and discussion

**Fig 3:**
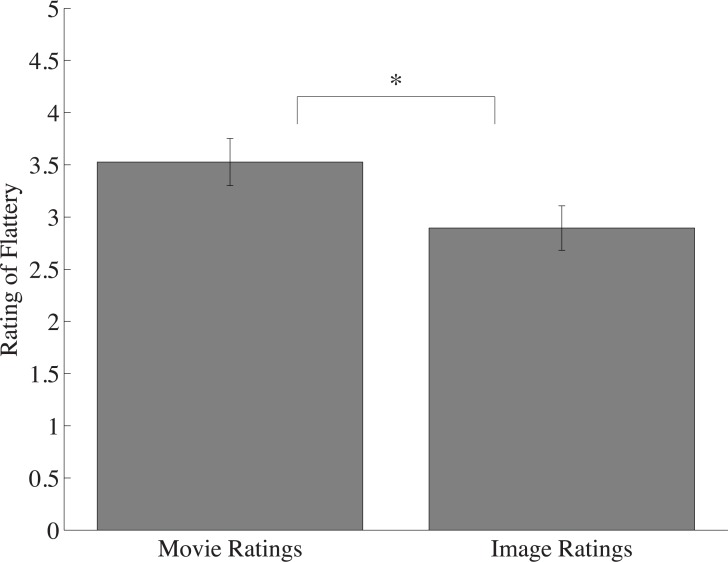
The average flattery ratings of all inverted movies compared inverted static images. Inverted movie ratings were significantly higher than their inverted component images. Error bars represent one standard error of the mean (SEM). Note: * denotes significance at p < .005.

One observer was removed from analysis due to not following task instructions. As with Experiment 1A, and consistent with the work of Post and colleagues^18^, inverted bodies in motion were viewed as more flattering than their constituent static images (t(17) = 5.66, p < 0.0001, *d* = 1.35; Fig 3). Furthermore, the magnitude of this effect was not significantly different for the inverted stimuli relative to the upright stimuli, as revealed by a between subjects t-test (M_FEupright_ = 0.85; M_FEinverted_ = 0.63; p > 0.05), suggesting the FE is not configurally dependent. It is possible the stimuli themselves, which depicted children at play in various positions and orientations (some sitting, some lying down), mitigated the configural dependence typically observed for bodies.

## Experiment 1C

It has previously been found that facial recognition is hindered when natural facial movement is disrupted [7, 13]. Similarly, disrupting coherent motion also reduces the Frozen Face Effect [18], suggesting that the benefit does not depend simply on a rapid succession of images or motion more generally. Experiment 1C explored whether coherent motion is driving the Frozen Effect observed in the first two experiments. To test this, observers evaluated the same upright images and movies from Experiment 1A, with the coherence of the depicted motion disrupted by scrambling the order of the frames in each movie. This isolated the role of coherent motion while controlling for other properties such as image speed, content, and number of images seen in a given unit of time.

### Method

#### Participants

Nineteen undergraduate students from the Rhodes College community, age 18–22, participants in Experiment 1C. All participants were naïve to the purpose of the experiment.

#### Stimuli and design

Stimuli were as described in Experiment 1A, except for the videos, which were individual frames presented in random order. Frame rate was the same as in previous experiments (30 fps), but the randomized presentation effectively broke motion continuity. All other design elements were as described in Experiment 1A.

#### Procedure

The experimental procedure was identical to that of Experiment 1A —participants provided flattery ratings for the scrambled videos and each of their component images.

### Results and discussion

**Fig 4:**
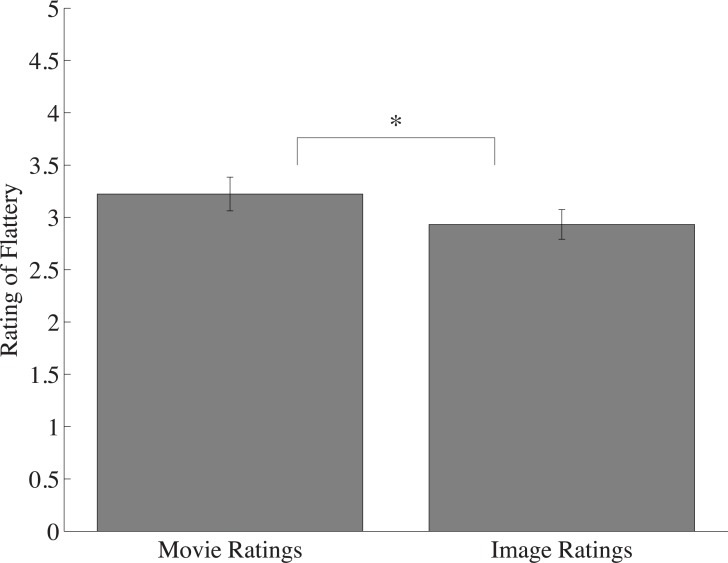
Average flattery ratings of all scrambled movies compared to static images. Scrambled movie ratings were significantly higher than their inverted component images. Error bars represent one standard error of the mean (SEM). Note: * denotes significance at p < .05.

Two participants were removed from analysis due to not following task instructions. A within-subjects t-test revealed that the videos, even in scrambled form, were viewed as significantly more flattering than their component images (M_movie_ = 3.22; M_image_ = 2.93; *t*(16) = 2.38; *p* = 0.03, *d* = 0.57; see Fig 4). However, the magnitude of this effect was significantly reduced relative to Experiment 1A —a between-subjects t-test showed that the flattery difference between the scrambled videos and component images was significantly smaller than the difference between the coherent motion videos and component images (i.e., the magnitude of the FE; M_intact_ = 0.86; M_scrambled_ = 0.29; *t*(31) = -3.12; *p* = 0.004, *d* = 1.1; Fig 4). These results suggest coherent motion plays a critical role in producing the Frozen Effect.

## Experiment 2

Experiment 2 explored whether the predictability of motion modulated the Frozen Effect. We hypothesized that bodies moving in a prototypical fashion (e.g., a dancer executing a pirouette), when a given movement conforms to a constrained and predictable space, might elicit an even stronger Frozen Effect than that observed in the first experiment.

The motion depicted in Experiment 1 was largely unpredictable—children moving about in non-prototypical fashion—and yet a significant preference for bodies in motion emerged. In Experiment 2, we selected stimuli containing prototypical motion (e.g., dancing, running, exercising, skating, piano playing, card shuffling, etc.), which might have allowed observers to formulate clearly defined expectations. We hypothesized that a more constrained motion space, with fewer degrees of freedom and stronger predictive contextual information, would increase the magnitude of the Frozen Effect.

### Methods

#### Participants

Eighteen undergraduate students from the Rhodes College community, age 18–22, participated in Experiment 2. All participants were naïve to the purposes of the experiment.

#### Stimuli and design

We created a stimulus set containing 25 1-second videos (30 fps; resolution 530 × 360) of individuals executing highly prototyped, predictable actions. Examples included depictions of dancing, figure skating, walking, and exercising. The face of the figure in each video was either obscured or out of frame. As in Experiment 1, stimuli were presented in random order.

#### Procedure

The experimental procedure was identical to that of Experiment 1A, except that videos disappeared upon their conclusion to limit the potential influence of the final frame on participant ratings.

### Results and discussion

**Fig 5:**
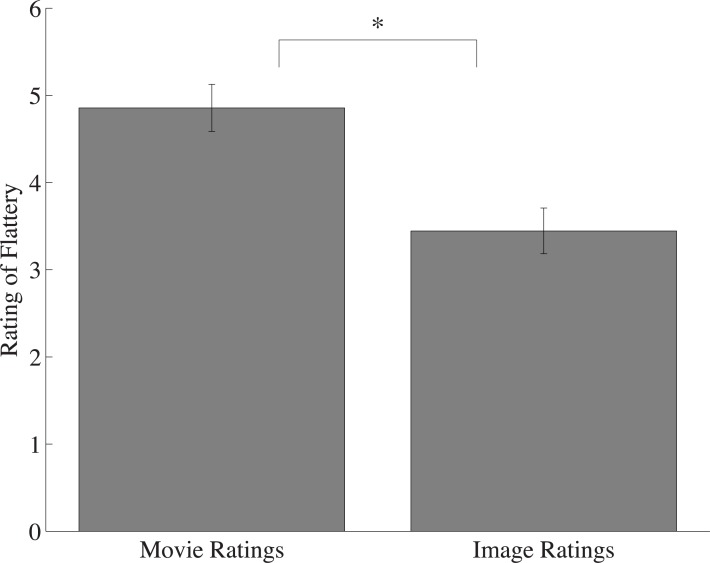
The average flattery ratings of all prototypical movies compared to static images. Movie ratings were significantly higher than their component images and this difference was significantly greater than the one observed for Experiment 1A. Error bars represent one standard error of the mean (SEM). Note: * denotes significance at p < .0005.

A within-subjects t-test replicated the results of Experiment 1A —participants overwhelmingly regarded the videos containing prototypical movements as more flattering than their static counterparts (M_movie_ = 4.86; M_image_ = 3.44; *t*(17) = 5.97; *p* < 0.0001, *d* = 1.43); Fig 5). Importantly, using prototypical videos increased the size of the Frozen Effect when compared to Experiment 1A (mean difference rating for Experiment 1A = 0.86, for Experiment 2 = 1.41), as revealed by a one-tailed, independent samples t-test (*t*(32) = 1.96, *p* = 0.03, *d* = 0.68). This suggests that bodies executing prototypical movements, which may be more predictable, are more flattering in motion than when viewed in static form, and this difference was enhanced relative to what was observed in Experiment 1A (where bodies in motion were more unpredictable).

## Experiment 3

Given that flattery ratings were enhanced for both faces [18] and bodies in motion, we tested whether this effect generalized to other object categories as well, specifically, to non-biological objects often viewed in motion. We created a set of videos depicting moving objects, for example, machines, modes of transportation, and toys. The motion depicted in this stimulus set is even more constrained and predictable than the prototypical bodies-in-motion stimuli from Experiment 2, as bodies have far greater degrees of freedom than objects with well-defined motion patterns (e.g., vehicles, machinery, and automated toys). This implementation allowed us to assess whether the Frozen Effect generalized beyond biological entities.

### Methods

#### Participants

Twenty-three undergraduate students from the Rhodes College community, age 18–22, participated in Experiment 3. All participants were naïve to the purposes of the experiment.

#### Stimuli and design

We created 29 1-second videos of objects typically observed in motion, such as machines, modes of transportation, and toys (resolution 540 × 360). Videos of objects commonly observed in motion were collected from YouTube. All videos selected were recorded from a stationary camera.

#### Procedure

The experimental procedure was identical to that of Experiment 1A —participants provided flattery ratings for the non-biological videos (i.e., 29 video ratings) and each of their component images (i.e., 870 static image ratings), for a total of 899 trials.

### Results and discussion

**Fig 6:**
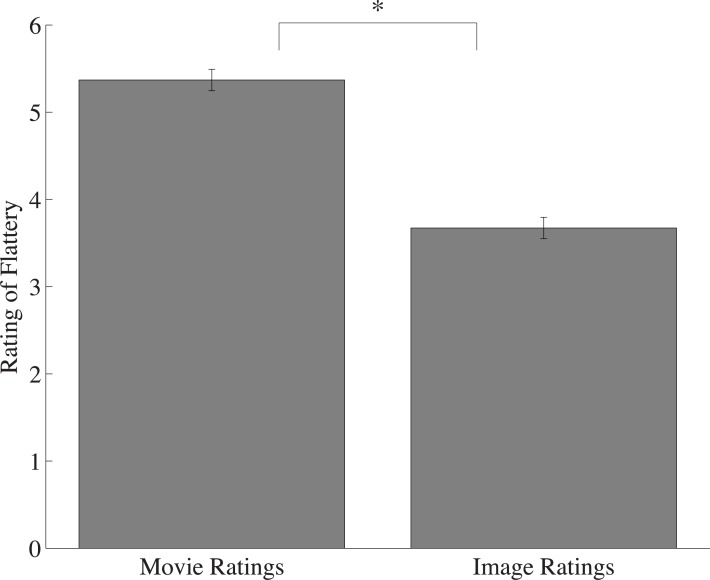
Average flattery ratings of objects in motion compared to static images. Movie ratings were significantly higher than their component images, and this difference was significantly greater than the one observed for Experiment 1A. Error bars represent one standard error of the mean (SEM). Note: * denotes significance at p < .0005.

Replicating the overall effects observed in previous experiments, observers rated the videos of objects in motion as more flattering than their component images (M_movie_ = 5.37; M_image_ = 3.67; *t*(22) = 13.15, *p* < 0.0001, *d* = 2.75; Fig 6). This suggests that the Frozen Effect is not limited to biological entities, and is a generalizable phenomenon. Objects in motion were seen as more flattering than static objects, even though the information content was identical.

We hypothesized that more prototypical types of movement, regardless of object category (e.g., face, bodies, objects), would reveal a larger Frozen Effect than less predictable kinds of motion. This was preliminarily confirmed in Experiment 2, where the Frozen Effect for constrained, more predictable motion (e.g., dancing, skateboarding) was larger than for unconstrained, unpredictable motion (e.g., children playing). We further confirm this with a one-way between-subjects ANOVA, examining the Frozen Effect with motion predictability as a factor (from most unpredictable, children playing, to most predictable, objects moving). The analysis revealed a significant effect of predictability (*F*(2, 54) = 5.87, *p* = 0.005, *η*^2^ = 0.18), with the effect being smallest for the least predictable motion (kids-at-play; M = 0.86), largest for the most predictable motion (objects; M = 1.70), and in between for some predictability (bodies-in-prototypical motion; M = 1.41). Thus, the fewer the degrees of freedom available for movement (i.e., predictable), the more appealing that category appears when in motion.

## Experiment 4A

In all previous experiments, the number of static images rated by participants was substantially greater than the number of videos rated (ratio of 30:1). In order to eliminate the possibility that participants were basing their flattering ratings on seeing a more novel stimulus (i.e., the video), the current experiment equated the overall number of static images and videos viewed by observers.

### Methods

#### Participants

Sixteen undergraduate students from the Rhodes College community, age 18–22, participated in Experiment 4A. All participants were naïve to the purposes of the experiment.

#### Stimuli and design

In order to equate the number of videos and images, we selected 10 representative ‘target’ videos from each previously utilized category (3 children at play, 2 prototypical body movements, and 5 object movements; resolution 720 × 480). Target videos from each category were selected at random from the previous sets. The target video ratings were compared to their component static images, as in previous experiments. The ratio of videos to images viewed was balanced by adding additional ‘filler,’ non-target videos to the stimulus set. Only target videos were analyzed.

**Fig 7:**
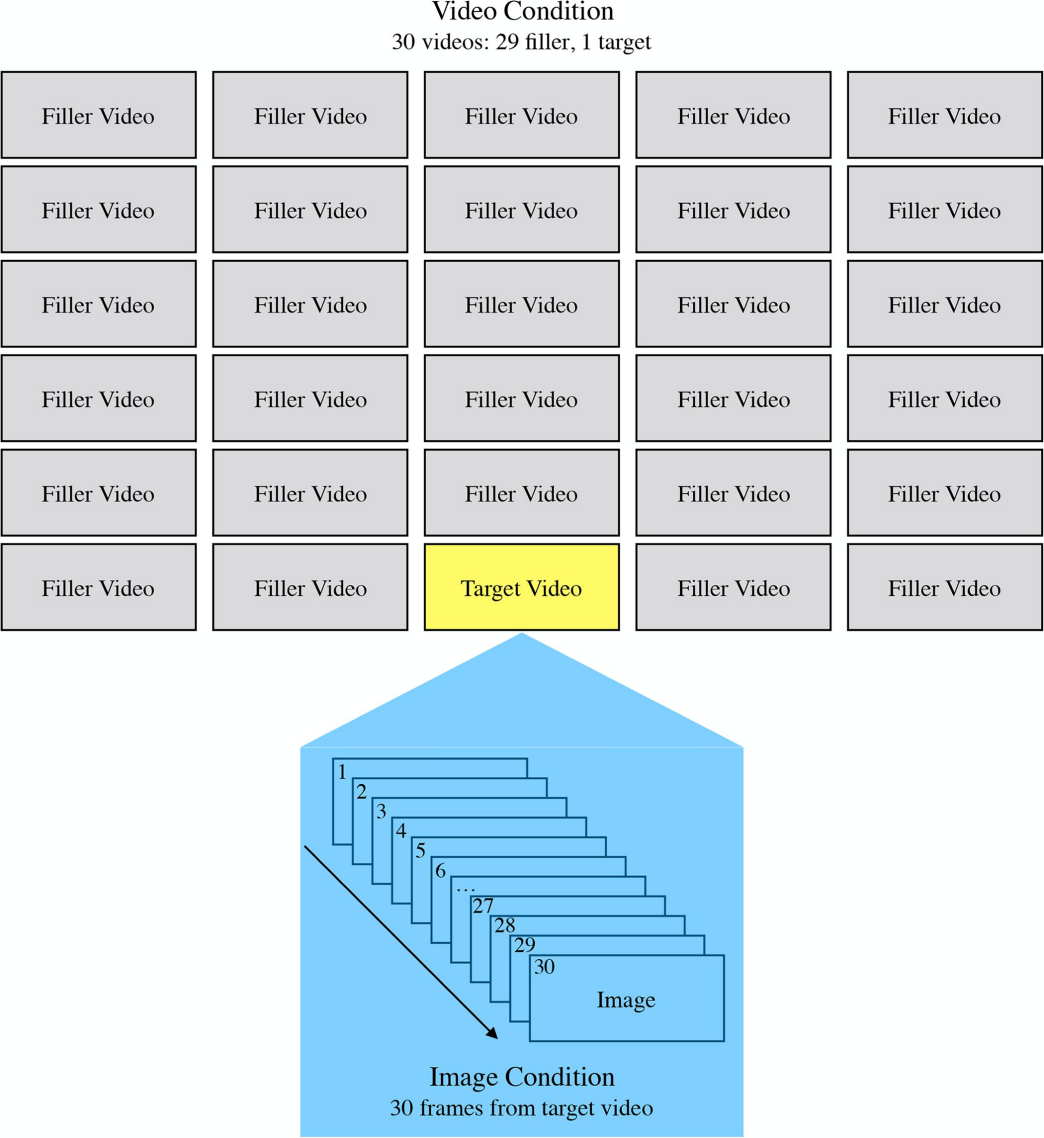
Schematic of the design for Experiment 4. (Top) A 1-sec target video was selected from a larger video sequence. Observers provided flattery ratings for all videos. (Bottom) Observers provided ratings for all static images from the target video.

Most of the target videos were 1-second clips taken from longer video sequences of the same subject (Fig 7). To balance the number of images and videos, these larger source videos were each segmented into 29 additional 1-second ‘filler’ videos. For target videos that did not come from a larger video clip, filler videos were from similar content, matched as closely as possible to the target video (e.g., target videos depicting airplanes were matched with similar looking videos depicting airplanes). As these target and filler videos came from the same or nearly identical source, the information content was well controlled. In addition to a given target video, observers rated 30 static images, taken from the target video, and the 29 filler videos. Half of the video set was composed of bodies, while the other half was composed of objects. By equating the overall number of static images and videos seen by each participant (i.e., 30 frames for each critical video and 30 videos containing similar content), we controlled for any effect of novelty in our previous experiments.

#### Procedure

The experiment proceeded as described in Experiment 1A, with observers giving flattery ratings for both videos and component static images. However, unlike previous experiments, stimuli spanned all prior categories and observers viewed an equal number of videos and images. The critical video ratings were once again compared to the average of their 30 constituent image ratings. Observers viewed a total of 300 videos (10 of which were critical, while the other 290 were filler used for counterbalancing purposes) and 300 static images, for a total of 600 trials.

### Results and discussion

**Fig 8:**
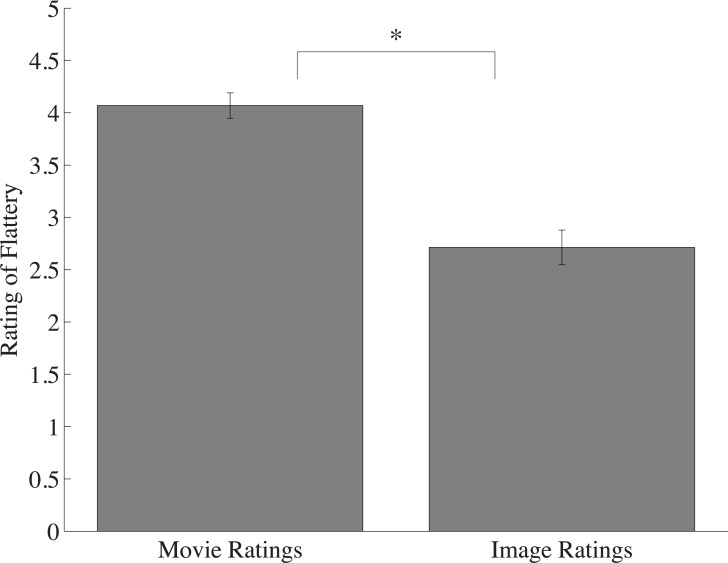
Average flattery ratings of objects in motion compared to static images when controlling for number of trials in each condition. Error bars represent one standard error of the mean (SEM). Note: * denotes significance at p < .0005.

**Fig 9:**
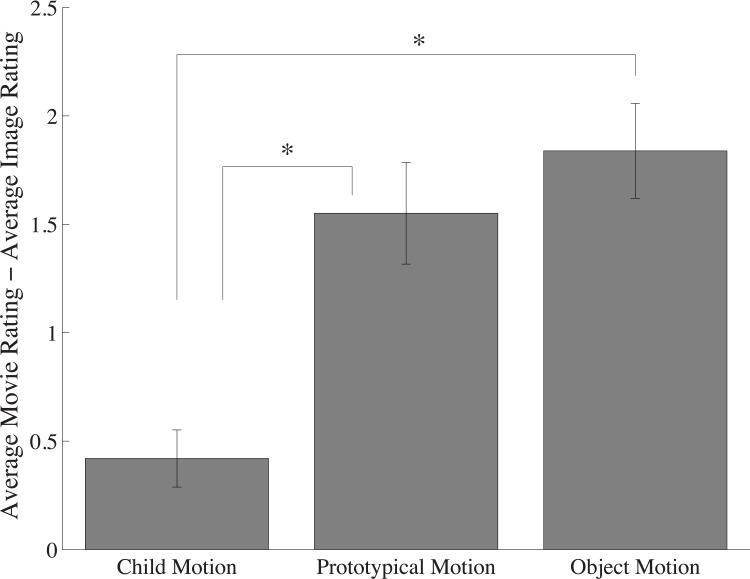
The magnitude of the Frozen Effect as a function of stimulus predictability. Error bars represent one standard error of the mean (SEM). Note: * denotes significance at p < .005.

A comparison of the flattery ratings for the critical videos and their corresponding component images once again revealed a significant Frozen Effect (t(15) = 9.12, p < 0.0001, *d* = 2.3), suggesting the difference in ratings is not due to the relative infrequency or novelty of videos (Fig 8). Additionally, as the critical videos spanned each category from previous experiments, we were able to replicate the prototypicality analysis described in Experiment 3. That is, we analyzed the magnitude of the Frozen Effect as a function of video category, which reflects predictability of the portrayed movement. A one-way, repeated measures ANOVA, revealed a significant effect of predictability (M_kids-at-play_ = .42; M_prototypical motion_ = 1.55; M_objects_ = 1.84; F(2, 30) = 18.72, p < 0.0001, *η*^2^ = 0.56), showing that, even within this small video sample size, predictability of movement strongly influenced the magnitude of the Frozen Effect (Fig 9).

## Experiment 4B

This experiment was a near complete replication of Experiment 4A, except that we equated the overall duration of the stimuli displayed on the screen. In Experiment 1, observers were exposed to the final frame of the vidoes for an extended period of time (until response), while in Experiments 2–4, the videos disappeared immediately upon completion while images remained on the screen until response. In this version, videos disappeared upon completion and images remained on the screen for one second before disappearing, which equated the overall amount of viewing time for each stimulus.

### Methods

#### Participants

Fifteen undergraduate students from the Rhodes College community, age 18–22, participated in Experiment 4B. All participants were naïve to the purposes of the experiment.

#### Stimuli, design, and procedure

All aspects of this experiment were identical to that of Experiment 4A except for the viewing time of the images, which disappeared from the screen after one second. This equated the overall viewing time of both images and videos.

### Results and discussion

Results closely replicate those of Experiment 4A. A robust Frozen Effect emerged even when stimulus durations were equated (*t*(14) = 6.26; *p* < 0.0001; *d* = 1.61), with a mean movie rating of 4.07 and mean constituent image rating of 2.73. Additionally, a strong effect of predictability once again emerged (F(2, 28) = 10.05; p < 0.001; *η*^2^ = 0.42).

## General discussion

These experiments suggest that the ‘Frozen Effect’ extends well beyond the category of faces, and applies to a variety of both animate and inanimate objects in motion. The extent of this effect correlates with the predictability of depicted motion. Observers rated the motion stimuli as more flattering than the identical information presented in static form, whether it was children-at-play, bodies in prototypical motion, or objects that one typically associates with movement. The ubiquity of this effect reveals the importance of dynamic motion when making aesthetic judgments. While this effect was first revealed using faces[18], clearly motion plays a critical role in perceptual preference across a range of visual domains. The systematic increase in the Frozen Effect as a function of predictability, the design of Experiment 4, and the overall brevity of experimental sessions (around 30 minutes) rule out the possibility that these results reflect participant boredom.

One explanation for the strength of this effect may be our limited exposure to static stimuli relative to stimuli in motion. Although the modern world has increasingly exposed us to static depictions (e.g., photographs) of ourselves, others, and objects, the majority of our visual experience remains that of near constant movement. Thus, differences in flattery ratings between moving and static stimuli may be driven in part by predictability of objects in motion. Our data suggest that the more constrained the movement space, the larger the Frozen Effect. Why might predictability create such a relationship? If one of the brain’s critical functions is to generate predictions (e.g., [30]), those predictions will be more accurate if the set of possible outcomes for a given input is small. Generating predictions from a constrained input set will reduce cognitive load relative to a more ambiguous input set (e.g., [31]), which could ultimately result in a more rewarding experience [32]. In other words, a moving dancer may be seen as more flattering than a static image of a dancer, given the challenge of predicting a particular outcome in the case of the latter stimulus.

These experiments extend the FFE[18] to multiple other object categories, leading us to propose a more generalized ‘Frozen Effect.’ In the original FFE study, authors speculated that the effect might arise due to temporal averaging [33, 34] of awkward transitions when viewing facial motion (e.g., blinking). While this effect may help to mitigate unseemly micro-moments in facial expression, this would also predict the greatest disparity in average image and movie ratings to occur in the ‘children-at-play’ category, where awkward moments would most predominantly arise (as opposed to objects-in-motion, where ostensibly there are fewer awkward transition periods). This was not the case however, as the ‘Frozen Effect’ was largest for objects-in-motion.

While the effects demonstrated here suggest motion is monotonically beneficial to aesthetic appeal, this may be true only for naturalistic stimuli. There are many examples in film in which predicted biological motion is violated, often to frightening effect. Motion, then, may be aesthetically beneficial only when it unfolds predictably. It may have the opposite effect when it deviates from the norm.

One interesting follow-up to these experiments might be to explore whether the FE extends to objects optimized for portraiture. Most of the movies used here were designed with motion in mind. However, would the FE remain if using stimuli that were originally conceived to capture a still-life, as might be true for a family portrait? Camera technology found in most cell phones today provides the option to acquire additional images on either side of the target image, which would be an easy solution to generating well-controlled stimuli.

Finally, these findings are particularly relevant in today’s image-conscious, social media culture, wherein individuals might display a carefully curated set of pictures to create a particular aesthetic. In light of these data, internet savvy social media and dating app users might be wise to incorporate animated GIFs, if possible (few platforms currently allow them as profile pictures), given the ostensible aesthetic benefit motion confers on a static image.

## Conclusions

Individuals rated bodies and objects in motion as more flattering than the identical information presented statically, something we refer to as the ‘Frozen Effect.’ This effect remained even when judging inverted stimuli, suggesting that an upright configuration of bodies and objects is not critical for eliciting the effect, consistent with the original Frozen Face Effect. When the motion of the stimulus was more predictable, the magnitude of the Frozen Effect increased. This may represent a kind of fault tolerance whereby bodies or objects with fewer constraints on the nature and direction of motion (e.g., children at play) do not appear aberrant. Bodies and objects that move in a prototypical fashion are more predictable, and therefore equivalent static depictions may be less aesthetically appealing.

## Ethics approval and consent to participate

All participants gave informed consent. This research, and all research described herein, was conducted in accordance with the Declaration of Helsinki and approved by the Institutional Review Board at Rhodes College.
